# Mutagenic joining of enzymatically induced DNA double-strand breaks, accompanied by persistent unrepaired DNA damage and a secretory protein phenotype, in HZE-exposed human cells

**DOI:** 10.1093/jrr/rrt169

**Published:** 2014-03

**Authors:** Zhentian Li, Farlyn Z. Hudson, Huichen Wang, Ya Wang, John P. Murnane, William S. Dynan

**Affiliations:** 1 Department of Radiation Oncology and Winship Cancer Institute, Emory University School of Medicine, Emory University, Atlanta, GA 30322, USA; 2 The State Key Laboratory Breeding Base of Basic Science of Stomatology (Hubei-MOST) and Key Laboratory of Oral Biomedicine Ministry of Education, School and Hospital of Stomatology, Wuhan University, Wuhan, Hubei 430079, PR China; 3 Institute of Molecular Medicine and Genetics, Georgia Regents University, Augusta, GA 30912, USA; 4 Department of Radiation Oncology, University of California-San Francisco, San Francisco, CA 94143, USA; 5 Department of Biochemistry, Emory University School of Medicine, Emory University, Atlanta, GA 30322, USA

**Keywords:** HZE radiation, DNA repair, secreted factor, stress response

## Abstract

High charge and energy (HZE) particles are a component of galactic cosmic rays. They cause complex damage to DNA and other cellular components, leading to both direct and indirect biological effects. Here, we investigate the hypothesis that one of these indirect effects is to compromise the accuracy of the DNA repair machinery, reducing the ability to cope with subsequent genotoxic insults. To this end, we used a new human reporter cell line with single-copy integrated fluorescent reporter cassettes that allow measurement of the frequency of mutagenic repair. Introduction of the rare cutting I-SceI nuclease stimulates both translocations (joining of two I-SceI sites on different chromosomes), and deletions (joining of two I-SceI sites after deletion of an intervening fragment) [ 1]. These can be measured simultaneously in the same cell population using different color reporter genes. To test the effect of HZE exposure on the frequency of translocations and deletions in this assay, cells were exposed to 600 MeV/u ^56^Fe ions or 1000 MeV/u ^48^Ti ions at doses of 0.3 or 1.0 Gy. They were allowed to recover and challenged with I-SceI at 1, 7, 14, 21 and 28 days post-irradiation. Results showed that HZE particle irradiation significantly increased the frequency of I-SceI translocations and deletions above baseline levels. There was an increase in translocations by up to threefold, seen in both ^56^Fe- and ^48^Ti-treated populations. There was also a more modest, but significant increase in I-SceI-mediated deletions seen in a population that received the higher dose (1.0 Gy) of ^56^Fe particles. The increased frequency of I-SceI-induced translocations and deletions persisted for 2–3 weeks with ^56^Fe (but not with ^48^Ti). The increased frequency of translocations and deletions was not observed in populations treated with low-LET radiation at doses up 3 Gy. Thus, the phenomenon, which we term the ‘mutagenic repair phenotype’ depends on both dose and radiation quality. The mutagenic repair phenotype was associated with an elevated frequency of micronuclei and excess DNA repair foci, suggesting that persistent genomic stress might be one causative factor [ 2].

To further explore the mechanism underlying the mutagenic DNA repair phenotype, we performed genome-wide expression profiling on cells that were harvested 7 days post-^56^Fe ion exposure. Results showed significant alterations in 234 genes. Many of the most highly induced genes encode secreted proteins that have previously been implicated in cellular senescence or pro-inflammatory processes. These findings suggest that a paracrine mechanism (induction of a set of senescence or inflammation-related proteins) may also contribute to the mutagenic repair phenotype.

Altered regulation of cellular double-strand break repair, with an increased reliance on an error-prone pathway, is a novel mechanism whereby an indirect effect of HZE exposure may amplify cancer risk.
